# Lymphocytic UV autofluorescence: A novel Ultraviolet-induced fluorescence dermoscopy finding in lichen nitidus—A series of two cases

**DOI:** 10.1016/j.jdcr.2025.10.064

**Published:** 2025-11-11

**Authors:** Varun H, Adarshlata Singh, Bhushan Madke, Meenakshi Chandak, Vangala Naga Nitya, Heeral Vadera

**Affiliations:** Department of Dermatology, Venereology and Leprosy, Datta Meghe Institute of Higher Education and Research, Jawaharlal Nehru Medical College, Wardha, Maharashtra, India

**Keywords:** Autofluorescence, Dermatoscopy, Dermoscopy, Lichen nitidus, Lymphocytes, Novel, Ultraviolet-induced fluorescence dermoscopy (UVFD)

## Introduction

Lichen nitidus (LN) is a rare, inflammatory papulosquamous dermatosis that predominantly affects children and young adults.^1^^,^^2^ While localized forms usually present with skin-colored to hypopigmented papules on flexural surfaces, generalized LN—characterized by widespread cutaneous involvement—is even rarer.

Although its etiopathogenesis remains unclear, dysregulated T-cell-mediated inflammation is hypothesized to drive the condition.^3^ Two-thirds of cases are asymptomatic, with spontaneous resolution typically occurring within a few months. Several clinical variants have been described, including localized, vesicular, palmoplantar, follicular, perforating, blaschkoid, linear and generalized.^4^

Herein, we report 2 pediatric cases of generalized LN demonstrating a novel Ultraviolet-Induced Fluorescence Dermoscopy (UVFD) finding—lymphocytic UV autofluorescence—with clinicopathologic correlation.

## Case presentation

### Case 1

**Fig 1:**
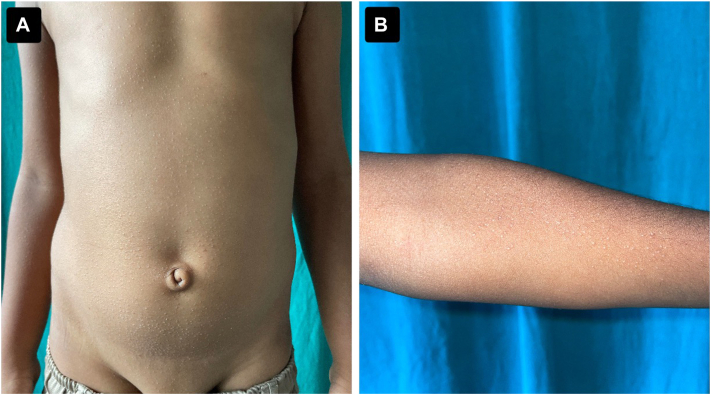
**A,** Clinical image depicting diffusely distributed, shiny papules all over the anterior trunk. **B,** Clinical image depicting the involvement of the flexural aspect of the forearm.

**Fig 2:**
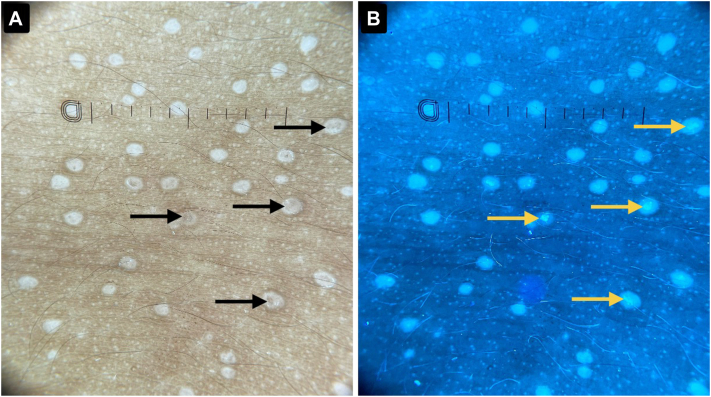
**A,** Contact dermoscopy in cross-polarized light (DermLite DL5, coupled with iPhone 12 camera) reveals well-defined hypopigmented clods with some containing central *brown shadows* (*black arrows*). **B,** UVFD (DermLite DL5 Wood-Mode 365 nm, coupled with iPhone 12 camera) of the same field exhibits *blue-white* fluorescence in the locations of the *brown shadows* (*yellow arrows*) (lymphocytic UV autofluorescence).

A 7-year-old boy presented to the dermatology clinic with a 2-month history of multiple, asymptomatic raised lesions affecting the trunk, neck, and both arms. There was no history of pruritus, pain, discharge, systemic symptoms (eg, fever), or drug intake prior to onset. Cutaneous examination revealed multiple, well-defined, discrete, hypopigmented to skin-colored papules distributed symmetrically across the trunk, neck, and bilateral upper limbs (Fig 1). Contact dermoscopy in cross-polarized light (DermLite DL-5) demonstrated well-defined hypopigmented clods with occasional peripheral scaling and focal brownish dots (Fig 2, *A*). Under UVFD (DermLite DL-5, wood-mode, 365 nm), these dots demonstrated paradoxical blue-white fluorescence, ruling out melanin deposition (Fig 2, *B*). The transition from cross-polarized to UVFD mode produced a dynamic blinking or winking appearance (Video 1, available on www.jaad.org), which we hypothesize reflects UV autofluorescence of the dermal lymphocytic infiltrate in LN.

**Fig 3:**
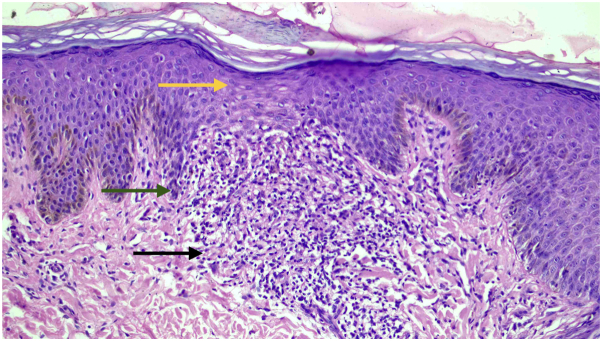
H&E-stained punch biopsy section demonstrating a ball-like lymphocytic infiltrate (*black arrow*) with elongated rete ridges attempting to enclose the lymphocytic ball (ball-and-claw appearance) (*green arrows*) along with focal epidermal thinning (*yellow arrow*) (10× ocular lens coupled with a 20× objective providing an effective magnification of 200×).

A 2-mm punch biopsy of a papule with the central brown dot revealed a thinned-out, parakeratotic epidermis with a hypomelanotic basal layer and vertically elongated rete ridges. These ridges were partially encircling a dense ball-like chronic inflammatory infiltrate at the dermo-epidermal junction, extending to the papillary dermis in the classic “ball-and-claw” configuration (Fig 3). Routine blood investigations were within normal limits.

Integrating the dermoscopic, UVFD, and histopathologic features confirmed a diagnosis of disseminated LN. Treatment with oral acitretin (0.5 mg/kg/d) and emollients led to complete clinical clearance after 4 weeks.

### Case 2

**Fig 4:**
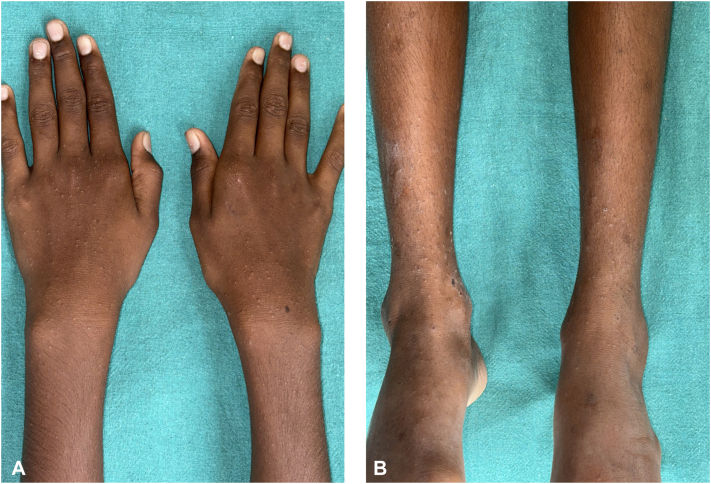
Clinical images depicting skin-colored papules involving the forearm **(A)** and the lower limb **(B)**.

**Fig 5:**
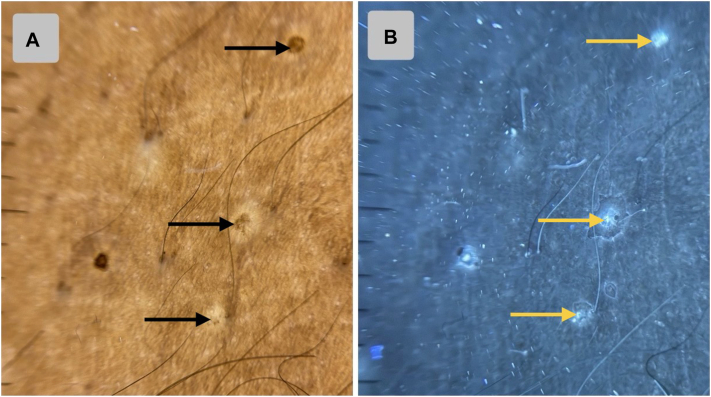
**A,** Contact dermoscopy in cross-polarized light (DermLite DL5, coupled with iPhone 12 camera) reveals ill-defined *brown* clods with a *whitish* halo (*black arrows*). **B,** UVFD (DermLite DL5 Wood-Mode 365 nm, coupled with iPhone 12 camera) of the same field depicting *blue-white* fluorescence in the locations of the *brown* clods (*yellow arrows*) (lymphocytic UV autofluorescence).

A 10-year-old boy presented with a 3-month history of pruritic, papular eruptions involving both upper and lower limbs. Examination revealed multiple, isolated, well-defined skin-colored papules with occasional crusting and excoriations distributed over the bilateral arms, forearms and legs (Fig 4). Contact dermoscopy (DermLite DL-5) demonstrated ill-defined brownish clods with a hypopigmented rim, which on UVFD (DermLite DL-5, 365 nm) displayed bright blue-white fluorescence consistent with lymphocytic UV autofluorescence (Fig 5). These findings supported a provisional diagnosis of LN; routine investigations were normal.

**Fig 6:**
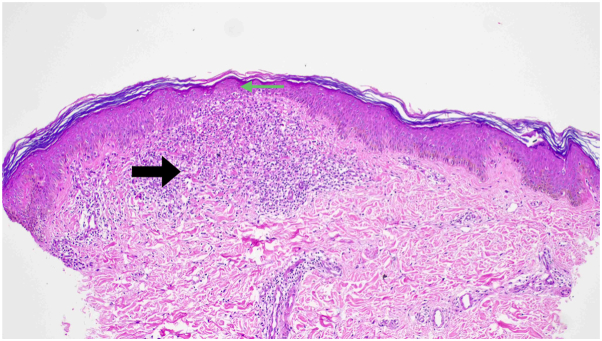
H&E-stained punch biopsy section demonstrating an ill-defined ball-like lymphocytic infiltrate at the dermo-epidermal junction (*black arrow*) underneath a hypomelanotic, atrophic epidermis (*green arrow*) (10× ocular lens coupled with a 20× objective providing an effective magnification of 200×).

A 2-mm punch biopsy revealed an ill-defined, ball-like lymphocytic infiltrate beneath a thinned, atrophic epidermis with unremarkable rete ridges (Fig 6). Based on the clinical, dermoscopic, and UVFD findings—including lymphocytic UV autofluorescence—along with histopathologic confirmation, a diagnosis of LN was established. Treatment with topical 0.1% tacrolimus, emollients, and antihistamines resulted in near-complete clearance of the lesions.

## Discussion

Dermoscopy of LN typically reveals well-circumscribed 1 to 2 mm hypopigmented clods with minimal scaling. Some lesions exhibit an ill-defined, central brown dot or shadow that can be mistaken for hyperpigmentation. Histopathologically, they correspond to the ball-like lymphocytic infiltrate beneath a thinned-out epidermis. This brown dot, considered a hallmark dermoscopic feature of LN, contrasts with the hypopigmented clods representing the epidermal acanthosis surrounding the infiltrate.^5^^,^^6^

UVFD operates through five fundamental interactions of UV light with the skin: reflection, absorption, scattering, penetration, and the Stokes shift phenomenon. These interactions depend on UV light’s engagement with cutaneous chromophores, namely keratin, melanin, and porphyrins, to generate distinct visual phenomena.^7^ In normal, non-pathogenic epidermis, UV light penetrates easily, producing minimal observable changes.

Hyperkeratotic and acanthotic epidermis, with thickened keratin layers, fluoresce blue-green to whitish under UVFD due to the Stokes shift phenomenon, reflecting keratin’s autofluorescence.^8^^,^^9^ In contrast, melanin strongly absorbs UV light and appears dark, aiding differentiation from pigmentary dermatoses.^10^

In our cases, the dermoscopic “hyperpigmented” brown dot showed direct histopathological correlation with a dense, ball-like lymphocytic infiltrate beneath a thinned, hypomelanotic epidermis, suggesting that the brown color seen under cross-polarized light arises from the infiltrate itself. On switching to UVFD, these brown dots did not darken as would be expected for melanin; instead, they fluoresced bright blue. Melanin was therefore effectively excluded as the source of the brown color, a conclusion supported by the histology demonstrating a hypomelanotic, atrophic epidermis corresponding to the site of the dot. Although keratin exhibits a blue-white autofluorescence, keratin is the unlikely explanation because the fluorescence is localized precisely to the brown dots, which are areas with an atrophic stratum corneum (which would have minimal keratin).

Activated lymphocytes and other inflammatory cells are well-documented to exhibit autofluorescence.^11^, ^12^, ^13^ We therefore hypothesize that the blue-white fluorescence observed via UVFD originates from the lymphocytic infiltrate rather than keratin, and melanin was excluded by its absence of UVFD-induced darkening and histopathology. Notably, when transitioning from cross-polarized to wood-mode (DermLite DL5), these aggregates dynamically “winked”–appearing brown in polarized light and fluorescing brightly in UVFD. We describe this optical phenomenon as lymphocytic UV autofluorescence in LN.

## Conclusion

These cases highlight the diagnostic synergy of dermoscopy and UVFD in LN. To our knowledge, this is the first report describing the lymphocytic UV autofluorescence—a dynamic, UVFD-specific phenomenon corresponding to lymphocytic infiltrates on histopathology. This observation may help differentiate inflammatory infiltrates from melanin, providing a non-invasive clue for the diagnosis of LN. Further studies are needed to validate the diagnostic utility and reproducibility of this finding in routine clinical practice. Standardizing Inflamoscopy (dermoscopy of inflammatory dermatoses) and UVFD could enhance non-invasive diagnostic approaches for papulosquamous disorders.

### Declaration of generative AI and AI-assisted technologies in the writing process

During the preparation of this work, the author(s) used Grammarly and ChatGPT in order to correct the grammar and improve the English of this manuscript. After using this tool/service, the author(s) reviewed and edited the content as needed and take(s) full responsibility for the content of the publication.

## Conflicts of interest

None disclosed.

## Supplementary data

Video E1
